# The Relation Between Subjective and Objective Measures of Visual Awareness: Current Evidence, Attempt of a Synthesis and Future Research Directions

**DOI:** 10.5334/joc.381

**Published:** 2024-07-18

**Authors:** Markus Kiefer, Thomas Kammer

**Affiliations:** 1 Department of Psychiatry, Ulm University, Germany

**Keywords:** visual masking, visual awareness, consciousness, awareness ratings, subjective measures, objective measures, psychophysics

## Abstract

Within the realm of consciousness research, different methods of measuring the content of visual awareness are used: On the one hand, subjective measures require a report of sensory experiences related to a stimulus. On the other hand, objective measures rely on the observer’s performance to accurately detect or discriminate the stimulus. The most appropriate measure of awareness is currently debated. To contribute to this debate, we review findings on the relation between subjective and objective measures of awareness. Although subjective measures sometimes lag behind objective measures, a substantial number of studies demonstrates a convergence of measures. Based on the reviewed studies, we identify five aspects relevant for achieving a convergence of measures. Future research could then identify and empirically test the boundary conditions, under which a convergence or divergence of subjective and measures of awareness is observed.

## 1. Introduction

Phenomenal consciousness (Block, 1995) denotes the experiential qualities of sensations from a first-person perspective (Nagel, 1974) such as the “redness” of a color or the “stinging” pain experience induced by heat. This private character of subjective experiences renders a reliable and valid assessment of this phenomenon difficult. Despite these problems, an adequate measurement of the content of phenomenal consciousness is necessary for its scientific investigation at the behavioral or neural level (Kiefer et al., 2011; Mashour, Roelfsema, Changeux, & Dehaene, 2020).

As one aspect of phenomenal consciousness, the content of visual awareness and associated mechanisms have been intensively studied in the past in neurologically intact observers (Kiefer & Kammer, 2017; Koster, Mattler, & Albrecht, 2020; Sandberg, Timmermans, Overgaard, & Cleeremans, 2010; Windey & Cleeremans, 2015) as well as in brain-damaged patients (Del Cul, Dehaene, Reyes, Bravo, & Slachevsky, 2009; Pöppel, Held, & Frost, 1973; Sahraie et al., 2006; Weiskrantz, 1997). Neurological intact observers are typically stimulated with briefly presented visual target stimuli, which are followed by a pattern mask consisting of for instance randomly arranged visual elements (e.g., Sandberg, Bibby, Timmermans, Cleeremans, & Overgaard, 2011; Sandberg et al., 2010). At short target-mask stimulus onset asynchronies (target-mask SOAs), observers have little or no awareness of the stimuli in most cases, whereas they are more likely aware of the stimuli at longer SOAs. In brain-damaged observers, awareness of visual stimuli is measured to determine residual vision and to isolate brain areas relevant for visual awareness (e.g., Del Cul et al., 2009; Garric et al., 2019; Sahraie et al., 2006).

Within this research domain, two distinct methodologies for probing visual awareness have been delineated (for recent discussions, see Jimenez, Prieto, Hinojosa, & Montoro, 2024; Schmidt & Biafora, 2024): Subjective and objective measures (Kiefer & Kammer, 2017; Overgaard & Mogensen, 2016; Ramsøy & Overgaard, 2004; Rausch, Muller, & Zehetleitner, 2015; Sandberg et al., 2011; Sandberg et al., 2010; Schmidt, 2015; Seth, Dienes, Cleeremans, Overgaard, & Pessoa, 2008). Subjective measures of visual awareness require observers to report their introspections related to sensory experiences elicited by the visual stimuli. Typically, observers categorically classify the percept as “seen” or “unseen” (Del Cul et al., 2009)) or perform graded ratings of the clarity of the percept (Ramsøy & Overgaard, 2004; Sergent & Dehaene, 2004) ranging from no sensory experience over experience of some stimulus features to a fully clear experience. Alternatively, subjective approaches prompt observers to use verbal phenomenological reports of the sensations to characterize the content of awareness in a fine-grained fashion (Albrecht & Mattler, 2012; Koster et al., 2020). Other widely applied subjective measures such as post-decisional wagering (Koch & Preuschoff, 2007) or confidence ratings (Rausch et al., 2015; Rausch & Zehetleitner, 2016; Sandberg et al., 2010) primarily capture meta-cognitive aspects of the phenomenal experience such as confidence in a decision (for a discussion, see Seth, 2008), but do not directly tackle the subjective content of awareness.

In contrast to subjective measures, objective measures of visual awareness are based on the observer’s performance and do not require report of introspections: Within a psychophysical approach, observers have to accurately detect or discriminate the visual stimulus in question across a series of trials (Eriksen, 1960; Kingdom & Prins, 2016; Schmidt & Vorberg, 2006). Besides directly analyzing performance accuracy, psychophysical indices putatively indexing visual awareness can be calculated from the accuracy distribution across trials (Asplund, Fougnie, Zughni, Martin, & Marois, 2014; Schmidt & Vorberg, 2006). For instance, sensitivity indices such as d’ based on signal detection theory can be determined from hit and false alarm rate to determine sensitivity independently from response bias (Green & Swets, 1966). Alternatively, psychophysical functions (Wichmann & Hill, 2001) can be fitted to the accuracy distribution, and thresholds as well as slopes of the psychophysical function are estimated to characterize awareness of the observers (Kiefer, Fruehauf, & Kammer, 2023a; Kiefer & Kammer, 2017; Koch & Preuschoff, 2007; Sandberg et al., 2011).

## 2. Are Subjective Measures a More Valid Index of Phenomenal Awareness than Objective Measures?

At a first glance, subjective measures of awareness such as ratings of the clarity of the percept seem to directly capture experiential qualities of sensations in line with the first person perspective of phenomenal consciousness (e.g., Overgaard, Rote, Mouridsen, & Ramsoy, 2006; Sergent & Dehaene, 2004; Seth et al., 2008; Windey, Gevers, & Cleeremans, 2013). As already explained above, phenomenal consciousness refers to the subjective perceptual experience of a stimulus (Block, 1995). Objective performance measures, in contrast, may not necessarily reflect the content of phenomenal consciousness. Instead, performance might merely index access consciousness, defined as a report or response in congruency with task instructions or action goals (Block, 1995). Access consciousness is characterized by information processing leading to an accurate report and involves a third person perspective. Performance might even not exclusively reflect access consciousness, but might be partially driven by unconscious processes (Koch & Preuschoff, 2007; Sandberg et al., 2011; Seth et al., 2008).

Furthermore, it has been argued that phenomenal consciousness is richer than access consciousness, the so-called phenomenal consciousness overflow hypothesis (Block, 2011, 2014). This hypothesis states that we are aware of more elements of a visual scene than we can report. If this phenomenal consciousness overflow hypothesis were correct, one could conclude that subjective measures, which capture phenomenal consciousness more directly by introspection, more validly characterize the full content of awareness than objective measures. A related argument is that subjective measures might be more exhaustive than objective measures, because not all perceptual experiences induced by a stimulus might contribute to performance (Jimenez, Villalba-Garcia, Luna, Hinojosa, & Montoro, 2019; Koster et al., 2020). Finally, unlike subjective measures, which provide information of awareness trial per trial, objective measures do not capture trial-wise fluctuations of awareness (Lähteenmäki, Hyönä, Koivisto, & Nummenmaa, 2015), because they are calculated on the accuracy distribution across a series of trials (Green & Swets, 1966; Wichmann & Hill, 2001).

However, subjective ratings of sensory experiences have also several limitations: Subjective ratings in general, including awareness ratings, might be influenced by response biases and do not necessarily index phenomenal experience (Asplund et al., 2014; Schmidt, 2015; Schmidt & Vorberg, 2006; Snodgrass & Shevrin, 2006). Ratings scales might also be used in a task-dependent fashion suggesting that the rating categories cannot be uniformly interpreted (Jimenez, Hinojosa, & Montoro, 2020; Jimenez et al., 2019). Furthermore, when using subjective ratings, observes might not specifically report the clarity of the visual experience, but also non-visual content such as intuitions, or confidence (Rausch et al., 2015; Rausch & Zehetleitner, 2016; Schmidt & Biafora, 2024; Seth, 2008; Zehetleitner & Rausch, 2013). Visual sensations induced by a masked stimulus may comprise experiences of movements, rotations or stimulus expansions (Albrecht & Mattler, 2012; Koster et al., 2020), which may not be precisely captured with a one-dimensional scale (Koster et al., 2020). Furthermore, as Overgaard (2018) pointed out, it can be questioned, whether the phenomenal overflow hypothesis is empirically supported: Frequently cited evidence such as the observation of change blindness (Rensink, O’Regan, & Clark, 1997) or Sperling’s experiments of iconic memory (Sperling, 1960) do not unequivocally support the notion of phenomenal overflow, because effects in these experiments can be alternatively interpreted. Hence, the overflow hypothesis cannot be taken as argument that subjective measures should preferred over objective measures to capture the richness of phenomenal awareness.

Finally, even more important, subjective measures, although based on introspections of sensations, rely on a report of the sensory experience. For instance, the widely used perceptual awareness scale (PAS) requires observers to judge their sensory experience on a four-point rating scale ranging from complete unawareness to full awareness (Overgaard et al., 2006; Ramsøy & Overgaard, 2004): 1 = “I do not see the stimulus at all”; 2 = “I saw a glimpse of something, but don’t know what it was”; 3 = “I saw something, and I think I can determine what it was”, 4 = “I saw the stimulus clearly”. Given that the sensory experience have to be translated into a report, subjective measures such as PAS ratings might similarly relate to access consciousness as objective measures and may not be considered as a more direct reflection of phenomenal consciousness (Persuh, 2018; Schmidt & Biafora, 2024). Interestingly, some scholars (Szczepanowski & Pessoa, 2007) even argued that objective measures more directly reflect phenomenal consciousness, whereas subjective measures index access consciousness, because they require meta-cognitive evaluations of the phenomenal content (see also, Seth, 2008).

In support of the proposal that subjective measures require meta-cognitive evaluations, the neural correlates of subjective vs. objective measures can dissociate. For instance, using measurements of oscillatory brain activity with the electroencephalogram (EEG), modulation of α frequency in the EEG was related to objective performance (Di Gregorio et al., 2022; Trajkovic, Di Gregorio, Avenanti, Thut, & Romei, 2023). In contrast, modulation of α amplitude was related to metacognitive efficiency defined as the relation of confidence ratings to objective performance. Furthermore, transcranial magnetic stimulation aimed at enhancing back-projections from motion-sensitive areas to lower-level visual cortex improved motion discrimination performance without affecting metacognitive efficiency (Di Luzio, Tarasi, Silvanto, Avenanti, & Romei, 2022). Conversely, enhancing back-projections from the intraparietal sulcus to lower-level visual cortex improved metacognitive efficiency without affecting motion sensitivity. This suggests that at least metacognitive efficiency, as a subjective measure of awareness, specifically recruits brain areas involved in perceptual decision making, over and above the areas that process the corresponding sensory feature (see also, Li, Hill, & He, 2014; Mazor, Dijkstra, & Fleming, 2022; Noy et al., 2015). It remains to be seen whether subjective measures other than confidence ratings, such as visibility ratings, also recruit brain areas related to perceptual decision making.

As an additional problematic aspect of subjective measures, it turned out that visibility ratings are not a neutral tool to assess trial-wise fluctuations of awareness within priming experiments: Performing visibility ratings in addition to the priming task abolished priming effects, attributed to the attentional demands inherent in this dual-task situation (Jimenez, Prieto, Gómez, Hinojosa, & Montoro, 2023; Kiefer, Harpaintner, Rohr, & Wentura, 2023b). At least within the context of priming research, the use of trial-wise visibility ratings cannot be recommended.

In conclusion, the arguments reviewed in this section suggest that subjective and objective measures have their specific limitations. In particular, despite their face validity, there is no agreement that subjective measures more directly reflect phenomenal consciousness. Hence, neither subjective nor objective measures seem to provide an intrinsically privileged index of visual awareness. As Schmidt and Biafora (2024) point out, subjective and objective measures differ only with regard to their task mode rather than intrinsically with regard to the probed content of awareness. As no optimal measure of visual awareness might exist for all purposes and situations, we believe that it is theoretically and empirically advantageous to elucidate the relation between these measures in different contexts and tasks. This may be then used as basis for a future theoretical and empirical analysis of the cognitive and neural processes given rise to responses in tasks, from which objective and subjective awareness measures are derived. Before we review findings in the upcoming sections of this article, we outline possible scenarios with regard to the relation between objective and subjective measures of visual awareness.

## 3. Possible Relations between Subjective and Objective Measures

As outlined above, there is considerable disagreement with regard to the type of representations captured by subjective and objective measures. While some scholars claim that subjective measures preferentially reflect phenomenal consciousness (e.g., Overgaard et al., 2006; Sergent & Dehaene, 2004; Seth et al., 2008; Windey et al., 2013), others propose the opposite and argue that subjective measures require meta-cognitive evaluations and therefore preferentially reflect access consciousness (Szczepanowski & Pessoa, 2007). As both subjective and objective measures require a report, it has been further suggested that both measures similarly reflect access consciousness (Persuh, 2018). Finally, it has been argued that objective measures, in contrast to subjective measure, might be more strongly influenced by unconscious processing (Koch & Preuschoff, 2007; Sandberg et al., 2011; Seth et al., 2008) and therefore do not qualify as exclusive measure of awareness.

**Figure 1:**
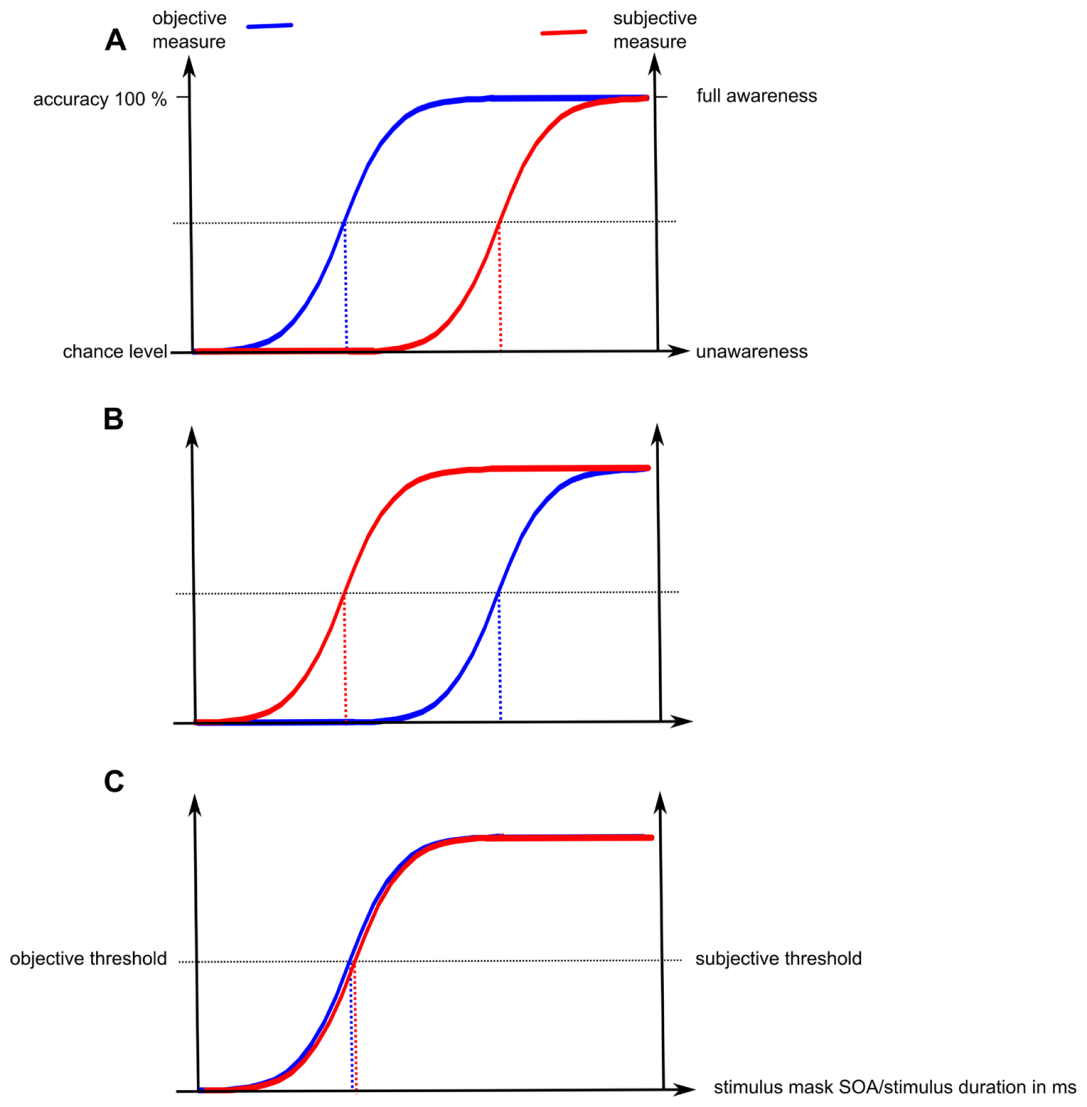
Possible relations between thresholds derived from subjective and objective measures of awareness. **(A)** Subjective measures lag behind objective measures. **(B)** Objective measures lag behind subjective measures. **(C)** Subjective and objective measures converge. Shown are hypothetical psychometric functions of objective (accuracy ranging from chance level to 100%) and subjective measures (subjective experience ranging from unawareness to full awareness) as a function of stimulus mask SOA or stimulus duration in ms. A gradual manipulation of stimulation mask SOA or stimulus duration leads to varying objective and subjective visibility. Subjective and objective thresholds (dotted lines) were determined at the inflection point of the respective psychometric function. For simplicity reasons, psychometric functions display the identical steepness. Abbreviations: SOA: stimulus onset asynchrony; ms: milliseconds.

Depending on these specific theoretical assumptions, three alternative hypothetical scenarios with regard to the relation between awareness thresholds derived from objective performance and subjective measures are possible (Figure 1): Firstly, objective and subjective measures could be related, because they in principle capture the same content of awareness, but subjective measures could be delayed by a constant lag. This lag may arise because objective performance might be partially based on fast unconscious processing, while subjective measures may require longer lasting visual consolidation giving rise to a specific phenomenal experience (Jimenez et al., 2019; Koch & Preuschoff, 2007; Sandberg et al., 2011). Alternatively, the temporal lag could also arise because only subjective, but not objective measures might depend on meta-cognitive evaluations (Seth, 2008), which includes an extra processing step. This relation between subjective and objective measures (i.e., absence of subjective awareness, but above-chance performance) characterizes blindsight phenomena in brain-damaged or neurologically intact observers (Koivisto & Neuvonen, 2020; Pöppel et al., 1973; Weiskrantz, 1997). Secondly, subjective and objective measures could be related, but subjective measures precede objective measures, because subjective measures capture are broader range of sensory experiences compared to objective measures. This phenomenon has been termed blindsense (Garric et al., 2019). Thirdly, subjective and objective measures could comparably reflect the content of visual awareness and therefore converge without exhibiting a lag in either direction (Kiefer et al., 2023a; Peters & Lau, 2015; Schmidt & Biafora, 2024).

## 4. Evidence on the Relations between Subjective and Objective Measures

In this section, we review findings on the relation between subjective and objective measures awareness and classify them according to one of the three scenarios described above. In our review, we include studies reporting subjective and objective measures of awareness in neurologically intact observes, in which stimulus visibility is varied by a visual masking procedure (Breitmeyer & Öğmen, 2006) or by employing an attentional blink paradigm (Pincham, Bowman, & Szucs, 2016). To make the picture complete, we also include studies in brain-damaged patients with impairments of visual awareness. Nevertheless, an exhaustive literature review is beyond the scope of the present article. Instead, we provide prototypical examples for the heterogonous and contradictory pattern of relations between subjective and objective measures of awareness (for discussions, see also, Jimenez et al., 2024; Schmidt & Biafora, 2024). To foreshadow the results of our review, the complex result pattern cannot be accounted for by any existing theoretical approach described above (for a general theoretical measurement framework, see Schmidt & Biafora, 2024). Nevertheless, we think that the heterogeneous pattern might be more than noise and could be informative with regard to the processing dynamics leading to subjective and objective measures under varying context conditions.

### 4.1. Thresholds of subjective measures lag behind those of objective measures

Subjective measures of awareness compared to objective measures may require longer lasting visual consolidation associated with phenomenal experience (Jimenez et al., 2019; Koch & Preuschoff, 2007; Sandberg et al., 2011) or might depend on post-perceptual meta-cognitive evaluations (Seth, 2008). Objective measure could partially depend on unconscious processing (Koch & Preuschoff, 2007). As consequence, awareness thresholds derived from subjective measures should be higher than those derived from objective measures. This includes the condition of above-chance objective performance (e.g., identification or discrimination) in the absence of subjective awareness. Such a dissociation between subjective awareness and objective performance is typically observed in the so-called blindsight phenomenon. Blindsight has been originally described in patients with damage to the primary visual cortex, who occasionally exhibit above-chance performance in identification or simple discrimination tasks for subjectively unaware stimuli presented to their “blind” visual field (Pöppel et al., 1973; Weiskrantz, 1997). Although the spared residual visual capacities in the impaired visual field have been sometimes underestimated (Sahraie et al., 2006), blindsight has been typically interpreted to reflect intact unconscious processing, as indexed by above-chance performance, but impaired conscious processing, as indexed by either an entire absence of subjective awareness (Type I Blindsight) or by reduced subjective awareness (feeling of a glimpse without experience of a perceptual content, Type II Blindsight).

Blindsight phenomena have been occasionally also observed in neurologically intact observers: Discrimination accuracy for visually masked stimuli has been found above-chance level, although observers reported to be subjectively unaware of the stimuli experience (Pournaghdali, Schwartz, Hays, & Soto, 2023; Song & Yao, 2016; Szczepanowski & Pessoa, 2007). Similarly, during an attentional blink paradigm (Raymond, Shapiro, & Arnell, 1992), in which successful identification of a first target impairs subsequent identification of a second target within a series of rapidly presented stimuli, identification accuracy was high, although rated subjective visibility of this stimulus was low (Pincham et al., 2016). Pincham and colleagues (2016) termed this phenomenon “experiential blink”.

However, these studies did not clearly differentiate in their subjective measures between states of complete subjective unawareness or states of feelings of a glimpse without experience of content. When differentiating between these two states of (un)awareness using PAS ratings (Koivisto & Neuvonen, 2020), subjectively complete unaware stimuli (Type I masked blindsight) were discriminated above-chance level only under certain masking conditions, whereas feelings of a glimpse (Type II masked blindsight) were consistently associated with above-chance level discrimination (for similar results, see Jimenez et al., 2019; Lähteenmäki et al., 2015). Nevertheless, independent of the type of blindsight, such demonstrations of blindsight in brain-damaged or neurologically intact observers suggest above-chance objective performance during complete or partial states of unawareness.

The existence of a temporal lag between subjective and objective measures as potential source of blindsight in neurologically intact observers (Koch & Preuschoff, 2007) was formally demonstrated by Sandberg and colleagues (2011). The authors conducted a reanalysis of data from a previous study (Sandberg et al., 2010) by determining subjective and objective awareness thresholds based upon fitted psychometric functions. In this earlier study, participants were presented with brief images of four simple geometrical shapes (circle, square, triangle, or diamond) at 12 different durations, ranging from 16 to 192 milliseconds in 16-millisecond increments. Following the presentation of the shapes, a mask containing all four shapes was shown. Participants were instructed to identify the displayed shape as quickly and accurately as possible, with an emphasis on accuracy over speed. Subsequently, participants indicated their subjective awareness using either PAS, a confidence rating scale, or a post-decision wagering scale. In their reanalysis, Sandberg and colleagues (2011) fitted psychometric functions to both objective performance accuracy and subjective awareness ratings across different stimulus durations (which also corresponded to changes in mask stimulus onset asynchronies). They consistently observed a horizontal shift between the accuracy curves and the curves based on subjective awareness ratings, with the largest shifts occurring in the PAS curves (Sandberg et al., 2011). In line with this, estimated subjective awareness thresholds, including those derived from PAS ratings, were higher, i.e., they emerged at longer stimulus durations, compared to the objective thresholds based on performance accuracy. As we discuss in the last section of this article, this interpretation crucially depends on a specific procedure of determining thresholds, which can be critically debated.

### 4.2. Thresholds of objective measures lag behind those of subjective measures

Albeit cases of blindsight in patients with damage to visual cortex are far more frequent, there is one report of the opposite phenomenon termed “blindsense”, that is patients are subjectively aware of a stimulus, but exhibit chance-level discrimination performance (Garric et al., 2019). Garric and colleagues (2019) investigated subjective awareness as well as detection and identification performance in seventeen patients with unilateral occipital lesions and accompanying deficits in one visual field. To assess subjective awareness, the authors modified the wording of the PAS and included an additional level that does not refer to visual experience. This Sensation Awareness Scale (SAS) comprises the following experiential categories: [1] I did not see anything; [2] I don’t think that I saw anything, but I am not sure; [3] I felt something, [4] I saw something, [5] I clearly saw something and can identify it. A subjective sensitivity measure was calculated using the area under the receiver operating curve indicating, whether the use of the SAS reliably differentiates between the presence and the absence of a target. Despite their inability to identify the stimulus during the objective forced-choice task, four patients demonstrated sensitivity to its presence on the subjective scale, surpassing chance levels. (Garric et al., 2019). Three out of these four patients also exhibited chance-level performance in the detection task. Given its independence of behavioral performance, Garric and colleagues (2019) assume that blindsense patients do not exhibit normal degraded vision, but experience conscious non-visual sensations in response to a visual stimulation. This indicates that a wide range of sensory experiences including non-visual sensations might be mapped on the response categories of awareness rating scales (see also, Zehetleitner & Rausch, 2013). Most likely, these non-visual sensations do not support accurate performance in objective discrimination or identification tasks (for discussions, see Koster et al., 2020; Schmidt & Biafora, 2024). At present, comparable blindsense phenomena have not been found in neurologically intact observers during visual pattern masking or the attentional blink, but there are reports of such a pattern in single observers during metacontrast masking (Koster et al., 2020). It is possible that the presence of subjective awareness in the absence of above-chance objective performance is a rare condition, only occasional observable.

### 4.3. Subjective and objective measures converge

Several studies indicate that subjective and objective measures similarly capture the content of awareness. Comparable subjective and objective measures of awareness were found in brain-damaged patients without blindsight or blindsense (Garric et al., 2019) as well as in neurologically intact observers during continuous flash suppression (Lamy, Alon, Carmel, & Shalev, 2015) and visual pattern masking (Jimenez et al., 2023; Kiefer et al., 2023b): Both measures similarly differentiated between stimulus unawareness and awareness. Peters and Lau (2015) specifically investigated the validity of the blindsight phenomenon in neurologically intact observers, i.e. above-chance level performance for subjective unaware masked stimuli, using a two-interval forced choice procedure to minimize response biases. The authors presented a left- or right-oriented Gabor patch within a series of forward and backward masks near threshold in one of two presentation intervals. Observers had to report for each interval the orientation of the patch (objective measure) and to bet on the decision of each interval as subjective confidence measure. Based on a Bayesian ideal observer model, their analysis revealed similar awareness thresholds for subjective and objective measures. Hence, Peters and Lau (2015) did not find evidence for blindsight in neurologically interact observers, that is above-chance performance in the absence of subjective awareness.

Studies that systematically manipulate stimulus visibility from complete invisibility to full visibility, such as by parametrically adjusting stimulus mask onset asynchrony (SOA), offer particularly valuable insights into the alignment of subjective and objective measures of awareness. This type of studies allows to test whether subjective visibility and objective performance follow a similar course as a function of the experimental visibility manipulation. By traversing through all states of awareness, from complete unawareness to full awareness, this approach provides a fine-grained tool for assessing the convergence of subjective and objective awareness measures.

During meta-contrast masking, objective stimulus discrimination (left vs. right pointing arrow) and visibility ratings on a PAS-like scale followed the same time course as a function of stimulus-mask SOA (Lamy, Carmel, & Peremen, 2017): Both measures showed an identical increment of visibility with increasing SOA.

By applying transcranial magnetic stimulation pulses at different SOAs, de Graaf and colleagues (2012) investigated the time course of awareness of left vs. right oriented gratings and upright vs. inverted faces using subjective and objective measures. Objective performance was obtained during two discrimination tasks (left vs. right orientation, upright vs. inverted faces). Subjective visibility ratings were performed using an in-house four-point rating scale with the following categories: [1] “I didn‘t see the grating orientation/potential face inversion at all”; [2] “I don‘t think I saw the grating orientation/potential face inversion”; [3] “I think I did see the grating orientation/potential face inversion”; [4] “I saw the grating orientation/potential face inversion clearly”. De Graaf and colleagues (2012) found a close correspondence between visibility ratings and discrimination performance as a function of TMS pulse SOA for both gratings and faces.

**Figure 2:**
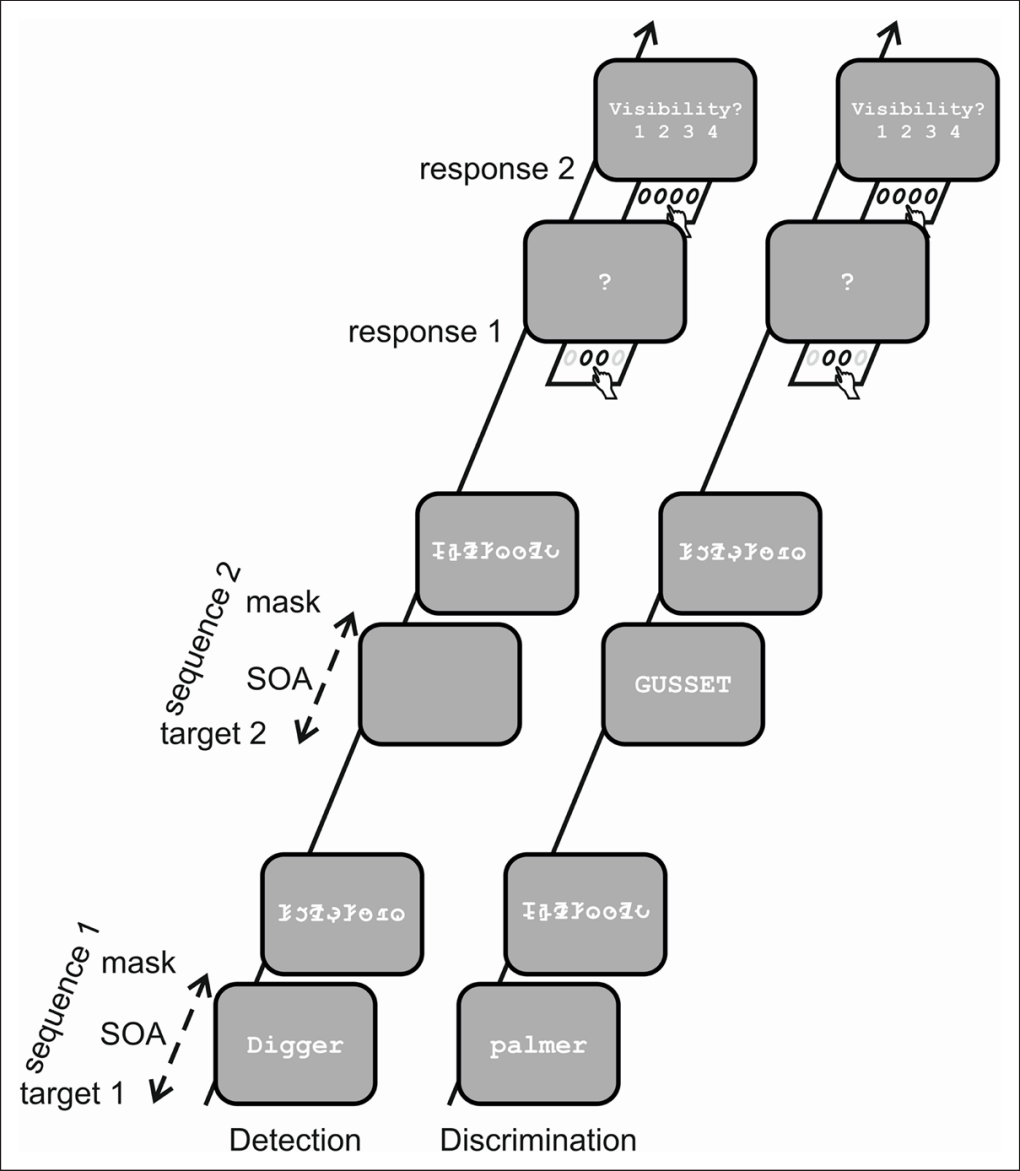
Temporal 2 alternative forced choice task. Detection and discrimination tasks comprised of two target-mask sequences with a target (one frame) followed by a mask formed by a string of false font letters (200 ms). Stimulus onset asynchrony (SOA) between target and mask adaptively varied in a range between 6.7 ms and 340 ms. The interval between the two sequences was 900 ms. After the second sequence, observers had to respond to the objective and subjective tasks, subsequently: (1) objective task: In what interval was the target flashed? (indicated by a question mark); (2) What was the visibility like? (PAS-scale, 1–4). In the detection task in one of the two sequences a word was flashed, the other sequence consisted of a mask only. The discrimination task consisted of two words, one written in capital letters, the other in small letters. This figure is adopted from Kiefer and Kammer (2023a) under the CC BY 4.0 license.

A recent study directly compared thresholds of psychometric functions obtained from subjective and objective awareness measurements across different stimulus feature and contrast levels (Kiefer et al., 2023a). In a two-interval temporal two-alternative forced choice (2-AFC) task (Figure 2), observers were presented with two stimulus-mask sequences. Within each pair, the task-relevant stimulus feature, such as the presence or absence of a stimulus, or the differentiation between uppercase and lowercase letters, appeared either in the first or the second interval. Following the second interval, observers were required to indicate the interval they believed contained the designated stimulus feature. This task minimizes contributions of unconscious processes to accurate performance.

Subsequent to the task response measuring objective performance, observers judged their sensory experience of the masked stimuli using PAS ratings. Prior to the main experiment, participants underwent rigorous training to ensure they could accurately align their phenomenological impressions with the PAS categories. This should enable observers to appropriately utilize the scale to report their sensations. Visibility of the word stimuli was manipulated and adapted to each observer by gradually varying the target stimulus mask SOA using a staircase algorithm. Threshold parameters of psychophysical functions fitted to the distribution of accuracy data and PAS ratings were determined for detection (stimulus presence) and discrimination (letter case) tasks encompassing high and low stimulus contrast. In analogy to the objective threshold definition of 0.75, i.e. the transition between pure guessing (0.5) and correct response (1.0) in the 2-AFC task, we defined the subjective thresholds from the fitted psychometric functions. Specifically, in the stimulus detection task, subjective thresholds were set at PAS level 1.5, indicating the transition between “nothing” and “glimpse.” In the stimulus discrimination task, subjective thresholds were set at PAS level 2.5, marking the transition between “glimpse” and “I saw something.”

Analysis revealed that both task type and contrast level significantly influenced awareness thresholds. Thresholds were higher for discrimination tasks compared to detection tasks, and for low contrast stimuli compared to high contrast stimuli. Moreover, task-related differences were more pronounced at low contrast levels than at high contrast levels. Remarkably, the mode of measurement (objective vs. subjective) did not influence the thresholds obtained. Indeed, thresholds across different conditions were nearly identical for both objective and subjective awareness assessments (Kiefer et al., 2023a). Consequently, subjective thresholds at the PAS level, reflecting the experience of stimulus presence without content, closely aligned with the objective detection performance threshold. Similarly, subjective thresholds at the PAS level, characterizing the visual experience of specific stimulus features, corresponded with objective discrimination performance. The convergence of thresholds was not accompanied by a convergence of the slopes of the fitted psychometric functions. In case of subjective ratings slopes tended to be shallower compared to objective ratings (for similar slope difference, see de Graaf et al., 2012). Nevertheless, this study (Kiefer et al., 2023a) and related work (de Graaf et al., 2012; Garric et al., 2019; Jimenez et al., 2023; Kiefer et al., 2023b; Lamy et al., 2015; Peters & Lau, 2015) demonstrate that objective performance measures and subjective ratings of visual experience can convey similar information on the feature-content of a percept.

## 5. Attempt of a Synthesis and Future Research Directions

As a theoretical starting point, we outlined three possible scenarios regarding the relation between subjective and objective measures of awareness: (i) subjective measures lag behind objective measures, (ii) objective measures lag behind subjective measures, (iii) subjective and objective measures converge. Although our literature review is certainly not exhaustive, we found exemplarily evidence for each of the outlined scenarios. Hence, there is no specific empirical support for only one scenario, but there is at least one piece of evidence for each of them. Admittedly, for the scenario, in which objective measures lag behind subjective measures, we were able to find only one supportive piece of evidence, the phenomenon of blindsense (Garric et al., 2019). This scenario appears to occur infrequently. Nevertheless, it does not seem to be the case, as is sometimes claimed, that subjective measures consistently lag behind objective measures. Instead, a substantial number of studies demonstrate a convergence of measures. This renders it unlikely that a valid assessment of visual awareness can only achieved by subjective measures. As we have argued above, subjective measures similar to objective measures capture access consciousness and not directly phenomenal consciousness, because even subjective measures require a report of the sensory experience, which includes a mapping of the experience on a sort of response scale (Schmidt & Biafora, 2024). It is therefore perhaps not too surprising that under certain yet to be specified conditions subjective and objective measures can provide similar information about the content of a percept. Based on the reviewed studies in the previous section, the following aspects might be important for achieving a convergence of measures:

- *Appropriate administration of the subjective visibility task*. Frequently, subjective measures rely on visibility ratings. Visibility ratings require an appropriate mapping of the sensory experiences to the different levels of the scale. In order to ensure a consistent, intended use of the scale, an intense training including feedback from the experiment can improve an accurate report of the quality of the sensory experience (for discussions, see Kiefer et al., 2023a; Kiefer et al., 2023b; Overgaard & Sandberg, 2021). Furthermore, the type of the visibility scale might be important: While there is an agreement that a dichotomous unseen/seen judgement leads to an invalid number of false unseen reports (e.g., Jimenez et al., 2024; Koivisto & Neuvonen, 2020), the optimal layout of the scale is discussed. A three-level PAS scale was associated with somewhat lower thresholds compared to the original four-level version (Sandberg et al., 2011). Furthermore, it is debated, whether the original categorical PAS (Ramsøy & Overgaard, 2004) or continuous PAS versions are optimal to report the varying experiential content of awareness (Wierzchoń, Anzulewicz, Hobot, Paulewicz, & Sackur, 2019). In sum, one can only expect a convergence of subjective and objective measures, if subjective measures are reliable and valid indices of the sensory experience.
- *Choice of the task for the objective measure*. To obtain objective accuracy measures of awareness a variety of tasks are administered such as detection (absence/presence of a stimulus) or discrimination tasks at various complexity levels (left/right orientation, letter case, shape, faces). However, detection thresholds are lower than discrimination thresholds (Kiefer et al., 2023a; Kiefer & Kammer, 2017). Furthermore, depending on the complexity of the feature to be discriminated, discrimination thresholds vary as well (Kiefer & Kammer, 2017). These differences in thresholds of objective performance for different tasks has consequence for their convergence on subjective levels of visibility. For instance, highly accurate detection performance is already achieved at PAS level 2 (glimpse without awareness of content), whereas highly accurate discrimination performance of letter case emerges at PAS level 3 (some stimulus impression) (Kiefer et al., 2023a). Consequently, in masking studies, for instance, detection performance reaches a ceiling in accuracy already at short stimulus mask SOAs (Kiefer et al., 2023a; Kiefer & Kammer, 2017), whereas subjective ratings require longer SOAs to reach levels of full visibility. A comparison of subjective and objective measure should therefore always consider the task-relevance of the rated sensory experience at a given subjective visibility level: A convergence of measures can only be expected, if the criterion content (Kahneman, 1968) or critical feature (Schmidt & Biafora, 2024) is the same for the subjective and objective tasks. Consequently, with regard to PAS ratings, an objective detection threshold should be compared to a subjective threshold at PAS level 1.5, i.e. between the experiential categories of “no impression” and “glimpse without awareness of content” (Kiefer et al., 2023a).
- *Choice of the subjective measure*. Although a majority studies employed visibility ratings related to the clarity of a percept, such as the PAS (Ramsøy & Overgaard, 2004), as subjective measure of awareness, confidence ratings (Zehetleitner & Rausch, 2013) or post-decisional wagering (Koch & Preuschoff, 2007) are also popular methods. It is apparent that the processes leading to the subjective reports in these various tasks are different. Confidence ratings, but also wagering do not require mapping a sensory experience on a scale, but involve meta-cognitive evaluations related to the sensory experience (Zehetleitner & Rausch, 2013). It turned out that thresholds based on confidence ratings were lower than those based on visibility ratings such as the PAS (Sandberg et al., 2011; Zehetleitner & Rausch, 2013). It is obvious that a possible lag between subjective and objective measures is larger for visibility ratings than for confidence ratings (Sandberg et al., 2011). Finally, as described above in point (i), when using visibility ratings, the precise layout of the scale might influence subjective thresholds.
- *Reduction of (unconscious) response tendencies*. It is possible that objective performance measures are partially influenced by unconscious response tendencies, leading to so-called response priming effects (Martens, Ansorge, & Kiefer 2011; Vorberg, Mattler, Heinecke, Schmidt, & Schwarzbach, 2003). Subjective measures may remain unaffected by unconscious influences, but may be contaminated by (conservative) response biases (Schmidt, 2015), for instance the tendency to be reluctant to report a glimpse, if there is only a weak sensory experience. Both types of response tendencies might result in a condition, in which subjective measures of awareness lag behind objective measures. It is therefore important to minimize unconscious biases on objective task performance. This goal can, for instance, be achieved by applying a temporal two-interval alternative forces choice task, which require a comparison of two stimulation intervals (Kiefer et al., 2023a). Furthermore, appropriate training of visibility ratings might reduce a tendency of a conservative scale use, which might induce an apparent divergence with objective measures. Finally, some response tendencies might be separated from perceptual sensitivity by choosing an appropriate psychometric measurement model, as argued in the next point (v).
- *Appropriate psychometric measurement model*. Although subjective measurements have some face validity, it cannot be taken for granted that the reported level of visibility, using the PAS scale for instance, truly reflects the sensory experience. A relatively high proportion of ratings indexing “glimpses” or “some impressions”, when actually no stimulus was presented in catch trials, illustrates this problem (Kiefer et al., 2023b; Lähteenmäki et al., 2015). This shows that it is necessary to apply a psychometric measurement model also to subjective measurements (Schmidt, 2015; Schmidt & Biafora, 2024). For instance, one can calculate subjective sensitivity measures in analogy to objective sensitivity measures based on signal detection theory (Green & Swets, 1966), which separates sensitivity from responses bias (e.g., reporting to have seen something, when actually no stimulus was present). In fact, objective and subjective sensitivity measures calculated according the principles of signal detection theory turned out to be quite comparable (Jimenez et al., 2023; Kiefer et al., 2023b). If thresholds are determined using fitted psychometric functions, the appropriate conditions for determining subjective and objective thresholds are important. In this context, the influence of performance and ratings values at the two poles of the psychometric function might have an important impact on thresholds. For instance, in the study by Sandberg and colleagues (2011), on the one hand, short stimulus durations resulted in accuracy responses at chance level in the objective task, while subjective ratings did not reach PAS level 1 (unseen) but remained between PAS 1 and PAS 2 (glimpse). On the other hand, long stimulus durations led to highly accurate responses (≥ 0.95), while subjective ratings did not reach PAS 4 (full awareness). It is known that thresholds as well as slopes critically depend on the two remaining parameters of a sigmoid function (γ and λ, lower and upper bound, Wichmann & Hill, 2001). Estimated subjective thresholds therefore might change with variations of experimental conditions, i.e. whether stimulation results in complete subjective invisibility at the lower pole of the psychometric function, or, as in the study by Sandberg and colleagues (2011) with residual subjective visibility at the lower pole.

At present, there is a lack of systematic research on the relationship between subjective and objective measures under different conditions. Future research could identify and empirically test the boundary conditions, under which a convergence and divergence of subjective and measures of awareness is observed. For instance, objective performance and subjective ratings thresholds could be obtained in a task condition, in which contribution of unconscious processing to objective task performance is more likely (e.g., responses to single stimuli in a classical 2-AFC task). Objective and subjective measures collected within a classical 2-AFC task could then be compared to a task condition, in which the contribution of unconscious processing to objective task performance is reduced (e.g., temporal 2-AFC task). One might expect a divergence of estimated subjective and objective thresholds under classical 2-AFC task conditions (i.e., subjective measures lag behind objective measures), while subjective and objective measure could converge under temporal 2-AFC task conditions. Similarly, studies could assess the influence of the amount of beforehand training of visibility ratings on subjective thresholds and their relation to objective thresholds. The five aspects proposed above might be an important starting point for achieving a convergence or divergence of measures and could be systematically investigated in future research. This kind of research could lead to empirically informed neuro-cognitive analyses of the processes and the neural substrate underlying responses in subjective and objective awareness tasks to guide a meaningful comparison of both measures (for recent examples, see Di Luzio et al., 2022; Mazor et al., 2022). In particular, such process analyses could provide a rationale for developing appropriate psychometric measurement models for objective and subjective awareness tasks in a theory-driven fashion (for a general framework, see for instance the Cue Set Theory proposed by Schmidt & Biafora, 2024).
